# Ochratoxin A in Portugal: A Review to Assess Human Exposure

**DOI:** 10.3390/toxins2061225

**Published:** 2010-06-01

**Authors:** Sofia C. Duarte, Angelina Pena, Celeste M. Lino

**Affiliations:** Group of Health Surveillance, Center of Pharmaceutical Studies, University of Coimbra, Health Sciences Campus, Azinhaga de Santa Comba, 3000-548 Coimbra, Portugal; Email: sofiacanceladuarte@gmail.com (S.C.D.); apena@ff.uc.pt (A.P.)

**Keywords:** ochratoxin A, Portugal, exposure, food, biomarker, cereal, bread, wine

## Abstract

In Portugal, the climate, dietary habits, and food contamination levels present the characteristics for higher population susceptibility to ochratoxin A (OTA), one of the known mycotoxins with the greatest public health and agro-economic importance. In this review, following a brief historical insight on OTA research, a summary of the available data on OTA occurrence in food (cereals, bread, wine, meat) and biological fluids (blood, urine) is made. With this data, an estimation of intake is made to ascertain and update the risk exposure estimation of the Portuguese population, in comparison to previous studies and other populations.

## 1. Introduction

**Figure 1:**
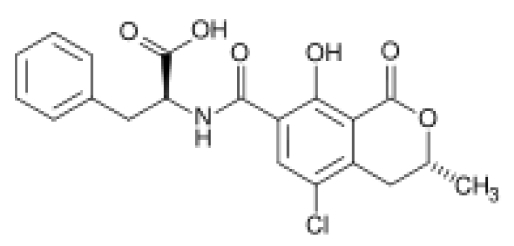
Chemical structure of ochratoxin A (*N*-{[(3*R*)-5-chloro-8-hydroxy-3-methyl-1-oxo-7-isochromanyl]-carbonyl}-3-phenyl-L-alanine).

The term mycotoxins refers to low molecular weight natural products, produced as secondary metabolites by filamentous fungi. It was coined in 1962 in the aftermath of an unusual veterinary crisis near London, England, during which approximately 100,000 turkey poults died. When this mysterious turkey X disease was linked to a peanut (groundnut) meal contaminated with secondary metabolites from *Aspergillus flavus* (aflatoxins), it sensitized scientists to the possibility that other unknown mold metabolites might be equally deadly. This triggered large-scale screenings targeted at mycotoxin discovery and identification, particularly between 1960 and 1975, named the “mycotoxin gold rush” [1]. Ochratoxin A (OTA; Figure 1) was discovered in this context, as a metabolite of *Aspergillus ochraceus* (hence its name) by van der Merwe and co-workers in 1965 [2] in South Africa, who isolated the toxic metabolite from corn meal intentionally inoculated with said fungus. Shortly thereafter, naturally occurring OTA was isolated for the first time from a commercial corn sample in the United States by Shotwell *et al.* [3]. In the same year of 1969 van Walbeek *et al.* [4] isolated the same mycotoxin from *Penicillium verrucosum*. Later, it was recognized as a secondary metabolite of several other *Aspergillus* and *Penicillium* spp. Although the widespread occurrence of ochratoxigenic species has been confirmed, each shows different behaviors in respect to ecological niches, the products (substrates) affected and their geographical occurrence [5].

OTA was described as one of the first group of fungal metabolites that are toxic to animals, which, with the aflatoxins, launched the distinctive and individualised science of mycotoxicology in the 1960s [6]. Even today, after the identification of more than 300 mycotoxins, OTA remains one of only about 20 mycotoxins known to occur in foodstuffs at sufficient levels and frequencies to cause food safety concerns [7]. 

As with other mycotoxins, the substrate on which the molds grow, as well as the moisture level, temperature, and presence of competitive microflora all interact to influence the level of toxin produced [1]. OTA has been found worldwide in cereals and its derived products [6,8], wine [9], beer [10], coffee [11], and other plant products [12]. It has also been detected in animal derived products, namely meat [13] and milk [14]. These chains of contamination (through both contaminated plant-based products and/or carry-over through contaminated derived products) reach up to humans [15], as OTA has been found in blood [16], urine [17], and human milk [18]. Thus, the main concern is the fact that human exposure to OTA is more likely to be from low level contamination of a wide range of different foods than from ingestion of a single, highly contaminated food product [19]. Furthermore, OTA is a moderately heat stable molecule that can persist through most food processing operations and, therefore, appears in final and derived products [5]. Although less mentioned, an inhalatory exposure route was also described and recently reviewed [20,21].

Regardless of the source of exposure, the primary target organ of this mycotoxin is the kidney. In addition to being a nephrotoxin, animal studies indicate that OTA is a liver toxin, an immune suppressant, a potent teratogen, and a carcinogen (group 2B) [15,22]. Ochratoxin A disturbs cellular physiology in multiple ways, but it seems that the primary effects are associated with the enzymes involved in phenylalanine metabolism, mostly by inhibiting the enzyme responsible for the synthesis of the phenylalanine_t_RNA complex. In addition, it inhibits mitochondrial ATP production, and stimulates lipid peroxidation [1]. 

OTA is believed to be responsible for an endemic porcine nephropathy described in the 80s in Denmark [23], and is implicated in endemic human nephropathies described in the Balkan region (Balkan Endemic Nephropathy) [24] and more recently in North African countries (Chronic Interstitial Nephropathy) [25]. However, OTA has also been described in non-endemic countries, like Portugal. In fact, the individual studies referenced below report OTA occurrence in Portugal, in both food and biological fluids. However, to ascertain the population exposure large-scale surveys, involving different groups of foods and different biomarkers, should be carried out. The absence of this type of contributions applied to the nation’s residents supported the idea of compiling this review. 

This manuscript provides an analysis of the OTA exposure of the Portuguese population through reported results on both occurrences of the mycotoxin in foodstuffs, as well as reported internal dose assessments. After the review of OTA external and internal doses, the most recent and representative data is used to draw a comparative contribution of each foodstuff to the OTA intake of Portuguese inhabitants. With this three-phase approach on the contributing factors: climate, food occurrence and dietary habits, an attempt is made to address the risk of OTA exposure in the Portuguese population. 

## 2. Portugal Portrayal

The Portuguese mainland occupies the western-most position in Europe, sharing a coastline with the Atlantic Ocean and a land frontier with Spain [26]. The mainland climate is typically Mediterranean, although the influence of factors such as the Atlantic Ocean and the landscape give to this small country climate contrasts since they provoke a degradation of the typically Mediterranean characteristics, so the Mediterranean climate loses its characteristics on the mainland from South to North and from the coast to the interior [27]. A Mediterranean climate is characterized by having a long, hot, and dry summer period, and a moderate winter, with a relatively low total atmospheric precipitation , while the Atlantic influence increases the overall humidity [26].

The climate is undoubtedly one of the main factors involved in mycotoxin contamination. In general, mycotoxins are climate dependent, plant- and storage-associated problems, and are influenced by non-infectious factors (e.g., bioavailability of (micro) nutrients and damage by insects and other pests), that are in turn driven by climatic conditions. Climate represents the key driving force of fungal colonization and mycotoxin production in the agricultural ecosystem [28]. 

Even though in Portugal the climatic conditions are favorable to ochratoxigenic mold development, and therefore to a higher OTA production risk, there is a lack of investigations related to OTA exposure. This stands out as a large fault, for several reasons; the Portuguese diet, in line with the traditional Mediterranean diet, presents a high consumption of cereals, with a second place in the EU ranking [29]. Since cereals are considered the main contributors to OTA exposure in Europe, the OTA exposure should merit more vigilance. Furthermore, the worldwide occurrence of the mycotoxin and the wide range of foods susceptible of being contaminated is important, even more so when considering that not all the foodstuffs consumed in Portugal are produced nationally, so the origin of OTA in Portugal can also be traced back to the countries from which the country imports food, because some of the extra-EU imports come from countries with no statutory and actions levels, nor awareness of OTA contamination. Finally, the recognized toxic effects should also contribute to a higher concern about this mycotoxin exposure. 

In Portugal, research on OTA started with a delay of 35 years after its first description in 1965, when OTA was reported for the first time in Portugal during a survey of wines [30]. Up until now, OTA research in Portugal addresses mainly the mycotoxin occurrence in food and beverages, complemented with bio-monitoring surveys, allowing some estimation of exposure. More recently an ongoing characterization of exposure through evaluation of bread and urine content has allowed some conclusions, although other undoubtedly contaminated foodstuffs, and thus also contributors to OTA intake, remain uninvestigated. Due to the historical and economical importance of wine in Portugal, surveys of mycobiota in vineyard environment and fate of OTA during wine making have also been carried out. 

## 3. Exposure Assessment

To provide the ultimate evidence that OTA exposure has taken place both testing the foodstuffs for occurrence and direct analysis of biological samples has been undertaken in Portugal. Below is a description of the research aiming to track exposure, through assessment of internal and external dose. 

### 3.1. Biological fluids contamination

In general, bio-monitoring is preferable over the evaluation of food contamination, given that variations in food preparation methods, food intake, contamination level, intestinal absorption, and toxin distribution and excretion lead to individual variations in exposure that are more readily measured with a biomarker [31,32]. In Portugal, only internal dose exposure biomarkers have been used as an indication of the occurrence and extent of exposure of the organism.

Considering blood specimen-derived OTA exposure assessments, the most intensively studied population has been the inhabitants of the central region. Serum analysis has been performed on 104 healthy inhabitants from Coimbra and two neighboring rural villages [33], resulting in a 100% positive series of results (LOQ = 0.05 ng/mL). No statistically significant differences were found between genders, although men presented consistently higher mean values (range 0.46–1.01 ng/mL) than women (range 0.38–0.60 ng/mL) in the three studied places. Furthermore, the two rural villages (0.78 ± 0.53 and 0.44 ± 0.31 ng/mL) presented higher values than the city of Coimbra (0.42 ± 0.18 ng/mL). Although this difference failed to reach significance, it is explainable through variations of climate and humidity levels, differences in consumption habits, namely alcoholic beverages, and ingestion of locally produced food, which significantly influences the OTA exposure [33,34].

OTA exposure of Coimbra inhabitants had already previously been assessed in a comparative testing against Aveiro inhabitants, although in this study all the individuals enrolled were undergoing haemodialysis [35]. OTA was detected in all the serum samples analyzed (LOQ = 0.05 ng/mL). The average values for the two cities were almost equivalent (0.5 ± 0.29 for Coimbra and 0.49 ± 0.22 ng/mL for Aveiro), as were the differences between genders in Coimbra. In Aveiro, men presented higher average levels (0.52 ± 0.24 ng/mL) than women (0.44 ± 0.18 ng/mL). 

It is worth mentioning that the mean levels encountered in Coimbra are similar, although slightly higher in the haemodialysed subjects (0.5 ± 0.29 ng/mL) [35] than in the healthy ones (0.42 ± 0.18 ng/mL) [33]. This fact may be a reflection of the effect of the dialysis treatment, resulting in the elimination of contaminants from the organism, whereas the slightly higher levels in the haemodialysed patients can be a result of a onetime diet exposure prior to sampling, a repeated exposure from a somewhat restricted therapeutic diet or from a blockage of the renal glucuronidation process [35,36]. 

When one compares the results of the healthy Portuguese population studied (Coimbra) with other healthy ones from other countries, similar degrees of exposure can be found. Exposure as measured by incidence was similar to Norway and Sweden [37], but when taking into account the average level, lower values were reported in places such as Zagreb (0.19 ng/mL) [38], Norway (0.18 ng/mL) and Sweden (0.21 ng/mL) [37], Lebanon (0.17 ng/mL) [39] and the Czech Republic (0.28 ng/mL) [40].

OTA bio-monitoring through blood presents undeniable advantages, one of them being the high levels encountered due to the long half-life, a consequence of its binding to plasma proteins, its entero-hepatic circulation and its renal re-absorption [2,34]. However, evidences show that it is a short-time marker and that it suffers from a high within-subject variability depending on season, which restricts its use at the individual level, besides involving an invasive collection procedure [16,41]. Therefore, more recently the urine biomarker has been gaining support and is regarded as a promising alternative, also because of proven better correlations with the level of consumption [17]. Still, the low OTA levels in urine require more sensitive and accurate analytical methodologies capable of offsetting the difficulties thus arising [42]. With that purpose, validation of a method for the determination of OTA in urine was conducted [43]. The methodology described therein was applied to 60 urine samples from Coimbra residents. The results revealed a 70% incidence, with mean value of 0.038 ng/mL and maximum value of 0.105 ng/mL (LOQ = 0.02 ng/mL). More studies on residents from the same city and also during winter ensued. The first described lower incidence (43%) and average (0.019 ng/mL) levels (LOQ = 0.007 ng/mL) [44]. The second described a similar frequency of contamination (73.3%), but a much lower average value (0.014 ± 0.007 ng/mL) [42]. If one tries to match these incidences with the weather conditions, the only pattern encountered is that the winter of 2005 (collection year of [44]) was the driest in 80 years, followed by the one of 2007 (collection year of [42]) and 2004 (collection year of [43]). During the winter of 2007, Coimbra’s incidence (73.3%) [42] resembled the one from Lisbon (70%) although the mean levels were not in harmony (0.014 ± 0.007 *vs.* 0.026 ± 0.017, respectively) [45]. However, and for the same period, the remaining regions analyzed (Bragança, Porto, Alentejo and Algarve) presented both a very high frequency of contamination (≈90%) and similar values for maximum concentration (ranging from 0.062 and 0.069 ng/mL). The average values were also higher, above 0.021 and up to 0.024 ng/mL [42].

In none of the studies conducted in Portugal to assess human exposure through OTA urine content [42,43,44,45] were significant differences between male- and female-provided samples detected. The only exception was the 20–39 years group of the study of Pena *et al.* [43], in which male-provided samples presented higher values of incidence and average level.

The incidence values (≈90%) reported in the Portugal national urine survey for OTA during the winter of 2007 [42] are among the highest when compared to the international ones. This comparison is retained for both endemic (Bulgaria—[46]) and non-endemic BEN (UK—[17]; Italy—[47]) areas. This similarity is not retained for the average value (0.022 ng/mL), for which the only lower value is from Hungary [48]. In fact, this is the only study whose results resemble the ones from Coimbra, both in incidence (61 *vs.* 73%) and mean value (0.013 *vs.* 0.014 ng/mL). 

When carefully evaluating and comparing the data presented herein, one can see that Coimbra exposure was amongst the highest (both in incidence and average value) when monitored through blood biomarkers [33], but one of the lowest when monitored through urine biomarkers [42]. It is important to underline however, that this comparison is made between studies carried out in different sampling groups, and different years, although during the same winter season. Furthermore, OTA in human blood samples is compromised by the long half-life of the toxin, so that a frequent dietary exposure will result in a steady state concentration [32,49]. So, the fact that the highest serum concentration is not related to the highest OTA consumption renders blood levels less useful as a biomarker of very recent exposure [32,46], whereas urine can thus present variations and reflect more readily recent higher or lower OTA intake. 

In conclusion, OTA and urine bio-monitoring contributed for the database of human exposure in Portugal, and showed a widespread prevalence of OTA contamination of the national population. Furthermore, the absence of persistent significant differences between most of the anthropometric parameters strongly suggests that the source of OTA is transversal to the population, which means that, considering that the major source of exposure is the ingestion of contaminated food, some common dietary foodstuff is probably implicated [50]. 

### 3.2. Food contamination

As such, further data was deemed necessary to glean information concerning the contamination levels of the most important foodstuffs that are both an integral part of the staple diet and with which high contamination levels were associated. According to the SCOOP report evaluation of OTA exposure in the European population, this would be cereals and their derivatives [51]. Accordingly, some nationwide surveys have also demonstrated the major contribution of cereals to OTA exposure, as in France [52]. 

If cereals and their derivatives are indicated as a susceptible food to ochratoxigenic fungi and hence OTA production, some authors suggest that if produced under organic agricultural practice the situation becomes worse due to the limited or restricted use of fungicides [53]. However, from the consumers’ point of view, organic products are perceived as healthier than conventionally produced ones. To ascertain the real risk for Portuguese and Spanish consumers, Juan *et al*. [54] analyzed 83 organic and non-organic cereal samples (45 from Valencia, Spain, and 38 from Coimbra, Portugal) including rice, wheat, barley, rye, oats, and maize commercialized in both countries during the winter of 2005. Statistically significant higher OTA contamination (LOQ = 0.19 ng/g) was detected in the Spanish samples, both in incidence (27% *vs.* 16%) and average level (0.93 *vs.* 0.64 ng/g). Regardless of the origin, 72% of OTA contaminated samples were organic and 28% were non-organic cereals. Furthermore, OTA frequency in whole-grain cereal was higher than in non-whole-grain cereal (33% *vs*. 14%) with the highest OTA level found pertaining to an organic sample of whole-grain rye (27 ng/g). Six samples of cereal grains, of which three rice samples with 5.90, 7.54 and 7.60 ng/g of OTA, two wheat samples with 7.6 and 7.97 ng/g, and a rye sample with 27.10 ng/g, surpassed the 5 ng/g EU maximum limit for cereal grains [55]. 

The incidence of contamination of rice (16.7%, 2/12; [54]) was comparable to the incidence previously found by Pena *et al*. [56]. Of the 42 rice samples analyzed, from different origins, OTA was only found in six (14%; LOD = 0.05 ng/g), with concentrations up to 3.52 ng/g (below the legal limit of 5 ng/g, [55]). Significant differences were found between samples of white rice and brown, basmati, aromatic and wild rice. None of the white rice samples contained OTA, but all of the brown, basmati, aromatic and wild rice samples analyzed showed contamination from OTA residues at detectable levels. An explanation for the difference in the contamination level observed exists only for wild rice. It is harvested at a higher moisture level, which is maintained for one or two weeks to accomplish fermentation, and because of a further stimulation of OTA production due to the high free amino acid content in wild rice, which possesses twice as much protein and amino acid than white rice [57].

When the most recent studies on OTA content of rice are compared, the low incidence of OTA in rice marketed in Portugal is evident, as compared to Vietnam (35%; [58]), Morocco (90%; [59]), Tunisia (25%; [60,61]) and Chile (42%; [62]). The same is true for the average level, since not even the maximum value detected in rice commercialized in the Portuguese market (3.52 ng/g) surpasses some of the average values found in Morocco (4.15 ng/g; [59]) and Tunisia (44 ng/g; [61]). 

However, most cereal grains are not consumed raw, as analyzed by Juan *et al*. [54]. They are further processed to different degrees into the most varied cereal derived products. During such food processing operations some losses of the moderately heat stable mycotoxin occur, the extension of which obviously influences the exposures of the final consumers. Thus, data from final cereal derived products, such as bread, is also necessary. In Portugal, bread is one of the most consumed foodstuffs. In 2005, 323,194 tons of bread were produced, of which 278,161 tons were wheat bread [63,64]. In fact, despite its small size, the country has different types of bread, wheat bread being the most consumed.

In the northern, interior Bragança district, inhabitants eat a traditional wheat bread, of about 0.5–1 kg of weight, baked in typical artisan wood ovens. Here there are no traditions of maize bread production and consumption. In the mostly urban Porto region, the locally made maize bread, Avintes *broa*, is distinguished and dominates over the conventional *broa* (maize bread). The first is bread made from a mixture of half maize and half rye flour, combined with hot water, salt and yeast (or as in the past with the last leavened dough), traditionally baked in a wood oven, with a cabbage leaf as the base support. It is distinguished from the conventional one not only by the flavor and dark color, given the added rye, but also by the soft and smooth texture and moist consistency. In the conventional *broa*, instead of rye, wheat flour is added. It is consumed not only in Porto, but also in the central region of Coimbra and in the capital city, Lisbon. Altogether, in these three regions, *broa* is the second most consumed bread type, after wheat. In the remaining regions of Alentejo and Algarve, *broa* is not traditionally consumed. Wheat bread remains as the most consumed, with Alentejano bread being a particularly famous and typical type, characterized by a traditional bread-making production, in which only wheat flour, water, salt and yeast are used. Another typical type of wheat bread is the Mafra bread, consumed specially in the capital region, with only wheat-based dough, water, salt and yeast. 

So, despite the general consensus that bread, as the major cereal derived product consumed, is probably the main contributor to OTA exposure, in assessing the OTA content of the different types of bread in Portugal a regional approach is encouraged due to the different bread make-ups. 

Just as for OTA bio-monitoring, the study of OTA content in bread started and has been more intensively carried out in the central region. The first study [65] was undertaken with the purpose of validating a HPLC-FD methodology for the determination of OTA maize bread (*broa*) content and was applied to 15 commercially available samples in the region of Coimbra. The frequency of contaminated samples was 60%, ranging from 0.033 to 2.65 ng/g (mean level 0.43 ± 0.9 ng/g). The following study [66], on the same region, comprised more samples (30 maize bread) and a more common type of bread (31 wheat breads). When considering the total samples, the frequency of detection was 40.9% (25) and the mean level 0.23 ± 0.86 ng/g. However, when the results are divided according to the type of bread, a higher level of contamination in maize bread as opposed to wheat bread, both in incidence (70% *vs.* 12.9%) and mean level (0.44 *vs.* 0.02 ng/g), is revealed. More recently, still in the same region, bread samples from the winter of 2007 were evaluated [67]. The maize bread samples persistently featured higher levels of contamination in relation to wheat bread, although these differences were not as marked as before, neither in frequency of detection (86.7% *vs.* 80%) nor in mean levels (0.48 ± 0.29 *vs.* 0.34 ± 0.34 ng/g). 

When comparing the contamination data of wheat bread in the two studies [66,67] an increase in the contamination figures is evident, whether measured by incidence or mean level. Amongst the possible reasons two emerge as the most important ones: the first due to contribution of different environmental conditions, namely during storage of the grain or flour. The second relates to different sampling criteria, since in the study of Duarte *et al*. [67], wheat bread samples also comprised bread made out mainly from wheat, but with the possibility of including different grains, seeds, fiber or bran. In theory, white flour for baking contains much lower concentrations than whole meal flour because the bran and offal containing high levels of OTA have been removed [5]. Moreover, since OTA is a moderately heat stable molecule that can persist after most food processing operations, including baking and, therefore, appear in final and derived products, it is reasonable to assume that the risk of presenting a higher OTA content is more likely for the whole-grain or fiber-enriched bread [68].

The study of Duarte *et al*. [67] also analyzed the OTA content of packed wheat bread. This novel segment of the bread-making industry showed exactly the same incidence as the normal (fresh) wheat bread, but a lower mean value (0.21 ± 0.13 ng/g). The difference in the mean value, although with no statistical significance (p = 0.134), is probably related to the higher content of preservatives in the latter, according to the label, and more intensive processing [67].

Dietary exposure to OTA varies considerably, depending on different factors, among which food-processing systems must be considered. These systems are often traditional and characteristic of the different geographical regions, which is also true for regional bread making practices [69]. These include not only the type of yeasts used and cereal grains included, the type of oven and temperature, but also the final features, namely a_w_ and pH, which might influence OTA content of the final bread products [70]. Furthermore, flour source and storage conditions have great weight in the OTA content of the finished product, and thus bread from different regions is likely to present wildly varying OTA content rates [71]. 

Thus, besides the region of Coimbra, during the winter of 2007, bread from other regions was studied in the attempt to portrait the region’s population’s risk of exposure through bread consumption.

Wheat bread from Porto showed a higher frequency of contamination than Coimbra (89.4% *vs.* 80%) although the average level was lower (0.19 *vs.* 0.34 ng/g). This situation is reversed when considering maize bread. Porto’s maize bread incidence of contamination is lower as the mean level (69.6% and 0.25 ng/g), when compared to Coimbra’s counterpart (86.7% and 0.48 ng/g). In both Porto and Coimbra a statistically significant difference was found between maize bread and wheat bread, as measured through mean levels (p value of 0.029 and 0.002, respectively). 

However, in Porto the traditional maize bread, Avintes *broa*, surpassed both incidence and mean level of common maize bread in either city, with statistical significance. The incidence almost reaches 96%, and the mean level 0.49 ng/g. This is probably due to the combination of maize and rye grains in their production, as these are two of the most contaminated cereals reported worldwide [67]. 

A study was also conducted in the Lisbon area, revealing that the incidence was high in both maize and wheat bread, and nearly all positive samples featured OTA content levels above the LOQ (0.1 ng/g). In both instances, values were slightly higher, though not significantly so, in maize bread. Similarly, average contamination levels were also more elevated in maize bread than in common wheat bread (0.28 *vs.* 0.21 ng/g). On the other hand, less than half of the Mafra bread samples contained detectable levels of OTA, and none of those featured OTA levels quantifiable through this method [45].

Bread from the winter of 2007 originating from Algarve and Bragança and its outskirts was also studied [71]. Algarve bread displayed higher incidence of contamination (80 *vs.* 65%) although a lower average value (0.2 *vs.* 0.3 ng/g). 

Considering the scarcity of data regarding OTA content in bread in foreign studies, comparisons are limited and difficult to make. Amongst the available studies, Spain [8] and Morocco [59] have particular relevance for comparison purposes due to the similarities with Portugal: they are located in the same general geographical region, both fall under the same climate class (Cs), and they are both in contact with the Mediterranean, which further influences climate [71,72]. With exception of the Mafra type of bread, all analyzed Portuguese wheat bread samples featured a higher frequency of contamination than Spain (19–20%) or Morocco (48%). The mean level was however several times lower than that of Morocco bread (13 ng/g). 

Concerning maize bread, the only foreign study found refers to samples collected in Turkey [73], which displayed an average value of 4.94 ± 0.20 ng/g, much higher than the mean levels in the Portuguese maize bread, *broa*, even considering Avintes maize bread. Although in the south-eastern part of Europe, Turkey also borders the Mediterranean, which influences climate. The samples where gathered in the Bursa region, which falls under the same climate class as Portugal, Cs [72]. Reasons for the evident discrepancy, if not satisfactorily related to climate, might be related not only to the agricultural practices, but also to the storage conditions of each country with further contribution from the type of bread-making process and type of cereals included [74]. 

In some studies [67,73], maize bread consistently presents higher levels when compared with wheat bread, which can be explained by the fact that, as demonstrated by Zummo and Scott [75], maize (*Zea mays*) has an ideal nutrient composition for fungal development and, therefore, one might consider that maize is probably more contaminated. Another reason in the case of Portuguese maize bread is the fact that it has an important difference in relation to wheat bread in the baking operation, since the dough from which it is made is less sieved than that for normal wheat bread. Therefore, it contains a lot of the outer layers of the grain, or bran, where in theory the mycotoxins tend to be concentrated [5]. 

However, other studies that reveal comparable contamination figures between them [45] or even higher for wheat [76] can also be explained by a theory that portions of glutamic acid are incorporated into OTA during its production, and so a high content of this amino acid in cereals, typical in wheat, could be a cofactor for the presence of OTA, as suggested by González-Osnaya *et al*. [74].

Returning to the Portuguese picture, it is important to underline that, in all instances and from all the data gathered since the first description of OTA in bread, which encompasses more than three hundred bread samples collected during the winters of the period between 2005 and 2007, only two samples surpassed the legal limit established (0.66%). To ensure the safety of consumers, this maximum permitted OTA level was set at 3 ng/g in cereal derived products like bread by the EC [55]. Both illegal referred samples corresponded to maize bread, one of them a common maize bread collected in Coimbra (5.86 ng/g; [66]) and the other Avintes maize bread collected in Porto (3.85 ng/g; [67]). 

Despite the fact that in the remaining bread samples (99.34%) the contamination levels remained far below the legal limit, and thus should pose no health risk, the occurrence of these high above legal limit samples, along with fairly high standard deviations signifies that OTA content values are distributed over a large range. Possible reasons include different origin of flour used to bake or greatly varying storage conditions, reasons which further emphasize the need for better surveillance.

Beyond bread making, the wine industry plays an important role in the Portuguese economy, which is one of the reasons to search for good quality standards in Portuguese wines. The main parameters of wine appreciation have been, throughout the years, color, flavor and taste. However, the development of new agricultural practices and the increasing sensitivity of analytical quality control methodologies, particularly associated to the enhancement of instrumental resolution which allows the detection of more of the so called trace compounds and in smaller concentrations, have been placing food safety as one of the main factors influencing the consumers’ opinion. Thus, marketing strategies also begin to take such variables into account. Amongst the often controlled trace compounds that may eventually act as contaminants and, therefore, be a source of concern to the consumer, is OTA [19,77]. This mycotoxin is nowadays globally considered the most relevant mycotoxin with respect to human health found in wine [77,78], where it was found for the first time in 1995 [9] 

OTA was first described in Portuguese wine [30], following a random sampling of commercially available Port wine (19 standard Port wines and 12 outstanding Port wines), adulteration of Port wine (3) and Vinho Verde (30). This survey attempted to characterize the OTA contamination profile of two of most known and consumed types of Portuguese wine: Port wine is a DOC fortified wine, whose fermentation is arrested with wine brandy, presenting an alcohol content between 19–22% and representing a blend of wines aged in oak; Vinho Verde is also a DOC wine, with an alcohol content between 9–11% and acidity above 6 g/L. OTA was detected only in the three Port wine adulterations, with levels up to 0.08 ng/mL (mean 0.063 ± 0.02 ng/mL). None of the Port wine and Vinho Verde contained detectable levels of OTA (LOD = 0.02 ng/mL). 

A more broad survey of wines from several Portuguese Demarcated regions (340 samples; (189 Port wine, 85 Vinho Verde and 66 wines from other regions) revealed that 69 samples (≈20%) had detectable levels of OTA [77]). No special incidence in any type of wine or region was noted. Only two of the 69 positive samples showed values above 0.5 µg/L, below the recommended maximum value of 2 µg/L of Office International de la Vigne et du Vin—OIV [79] and the EU legislation. 

In general, the southernmost regions of Europe present the highest levels of contamination [80], as compared to other worldwide winemaking regions, like South Africa [81,82], Hungary [83] or Turkey [84]. However, the results from the surveys on Portuguese wine [30,77], along with punctual results from other European southern countries like Spain [85], presented a rather low incidence (up to 20%) and mean levels below 0.5 µg/L. This can be due to specificities of the winemaking regions and different winemaking systems that determine different/lower OTA content in the final product, especially in the ones from Demarcated origin [30,85].

Furthermore, OTA occurrence in Portuguese sweet wines was assessed through a survey that included several countries, although insufficient and non representative samples prevent a characterization of the wine production of each country [86]. In the case of Portugal, of the nine samples analyzed, eight were positive (88.9%; LOD = 0.01 µg/L), with an average value of 0.06 µg/L and maximum value of 0.139 µg/L. Of the total worldwide sweet wine samples analyzed, 96.9% (281) were found to be positive, with a mean level of 0.499 µg/L. However, with the exceptions of Spain (represented by 186 samples) and France (represented by 49 samples), the rest of the countries surveyed, including Portugal were represented by sample size between one and nine, making any type of comparison difficult for several reasons, further aggravated by a lack of identification of regional origin and type of sweet wine. Therefore, as the authors stated, the influence of climatic conditions on the levels of OTA in wines may be responsible, at least in part, for the reported geographical differences among OTA levels in wines from different origins even in the same country, which renders global comparison of mean values among different countries a simplistic approach of limited value.

Since the first report of OTA in wines [9], the pressure for seeking food safety urged the OIV [79] to recommend a provisional maximum level in wine of 2 µg/L [19], latter adopted by EU [87] as the maximum level for red, white and rosé wine. However, values reported for wines above this limit are scarce and refer to southern Europe and North Africa regions (Mediterranean climates), that are more prone to contamination than those originating from central Europe [19]. There is no evidence that OTA levels are above 2 µg/L in the surveyed Portuguese wines, namely Port Wine and Vinho Verde [30,77].

The first and so far only study devoted to OTA contamination of baby and infant food marketed in Portugal is very recent [88] and was limited to a survey conducted in the Lisbon region between May and June 2007. The sample pool assembled consisted of cereal based items (flours, biscuits) and infant milk and follow-on milk (powder) intended for up to three year old infants. These 27 food samples randomly collected were of conventional (15) and organic (12) origin. 

In only three of the milk (powder) samples analyzed (two organic and one conventional) was OTA detected, and in only two of them (one organic and one conventional) was it quantifiable (LOQ = 0.028 ng/g), at 0.136 and 0.135 ng/g, respectively. It is noteworthy that the organic sample with quantifiable level was based on soy proteins and not common animal milk. The remaining products analyzed were cereal-based and consisted of one sample of biscuits/cookies and 19 types of cereal flours. The single sample of biscuits/cookies was positive (0.052 ng/g). It proceeded from organic production and in addition to cereal (60% wheat) included juice (28% grape juice). Both the organic production and the use of juices are considered risk factors for OTA occurrence [51]. Further interpretations can be erroneous since no other samples of biscuits were analyzed. Regarding the cereal flours investigated, OTA occurred in 12 (60.1%), although only seven (36.8%) were above the LOQ. The maximum contamination values were of 0.212 ng/g of organic origin and included beside the cereals (rice, oat and barley), apple, almonds and nuts. 

All results were below the maximum levels established in EU legislation (0.5 ng/g) for processed cereal-based foods and baby foods for infants and young children and infant formulae and follow-on formulae, including infant milk and follow-on milk [55].

In view of the results, the authors concluded that the occurrence of OTA detected in the infant food was not a public health problem. However, this small importance given is arguable. Although the encountered levels found were low, it is important to keep in mind that these commodities are among the first solid foods eaten by a vulnerable group, characterized by a higher consumption in relation to body weight and a somewhat restricted diet [5]. This is further corroborated by the recent health risk assessment of OTA by Kuiper-Goodman *et al*. [89], according to which infants from one to four years old were the only considered age-sex group that were went beyond the negligible cancer risk intake estimated. 

Foreign studies reporting OTA analysis in infant food are also scarce, and the majority has only been acquired as part of larger surveys of cereal foods in general [20]. However, data from the neighboring country Spain, reveal a similar incidence regarding cereal-based baby food (70%), with multi-cereals also being the more contaminated ones, with an incidence of 93% and an average level of 0.245 ng/g [90]. This pattern was not observed previously in Canada, where an evenly distributed occurrence among the oat (33.3%), barley (21.3%), soy-based (31.8%), and multi-grain (29.2%) cereals was reported. The average levels reported were also similar, ranging from 0.37 to 0.47 ng/g, with exception of barley that reached a mean value of 1 ng/g [91]. These incidence and average values are clearly higher than the ones reported in infant cereals in Turkey (16.7%; 0.221 ng/g; [92]) and Morocco (0%; [6]). 

Plant-based food, rather than food of animal origin, is considered to be the most important OTA source in the human diet. However, among food of animal origin, it has been shown that the carry-over of OTA from feed to animal products can occur in swine and poultry [93]. Swine and poultry diets are based on cereals and cereal by-products up to 50–60% on a dry matter basis, and these raw materials are the preferred substrate for *Penicillium* and *Aspergillus* growth [94]. Because of said carry-over effect, after gastrointestinal absorption, OTA is distributed via blood, mainly to the kidneys, and, in lower concentrations, to the liver, muscle and fat [95,96]. Even if the feed was contaminated with low levels, OTA residue occurrence in tissues is due to the high affinity of the toxin for proteins, namely albumin, which allows its accumulation in the organs of the animals [97]. Hence the pig tissue distribution pattern: blood > kidney > liver > muscle > fat [98,99]. Several studies worldwide demonstrated the occurrence of OTA in blood and edible organs.

The occurrence of OTA in meat was assessed a single time in the central region of Portugal [100]. The therein proposed method was applied to 12 chicken, 13 swine, and 13 turkey muscle samples. None of the analyzed chicken muscle samples contained positive levels of OTA (LOQ = 0.04 µg/kg), contrarily to the swine (7.7%) and turkey (30.8%) muscle samples, with average levels of 0.01 ng/g and 0.02 ng/g, respectively. The differences in OTA meat contamination did not reach significance.

The average value found in pork by this Portuguese study [100] was always at least ten times lower than the ones reported in other European studies, such as in Denmark (0.11 ng/g, [12]; 0.12 ng/g, [23]), and Romania (0.15 ng/g, [13]). In Italy [101] OTA was not detected in the 12 pig muscle samples analyzed, conversely to the 100% incidence detected in the same country by Matrella *et al*. [102]. In the later, all the 54 pigs, reared in different European countries and slaughtered in southern Italy, contained detectable levels of OTA, although these were very low, 0.024 ng/g. None of the samples exceeded the Italian Ministry of Health guideline value (1 ng/g), which means that, according to the authors, irrespective of the geographic provenance of pigs, OTA low incidence in pork and meat products is far from representing a real concern for consumers. This is probably the result of a worldwide improvement in feed quality due to the increased attention/control, at least in the period immediately before slaughtering. The authors concluded that from the consumer health point of view this means that the contribution from pig derived products to the total intake of the mycotoxin is negligibly small compared to other sources. OTA incidence in Portuguese pork (7.7%) was also inferior to the one detected in Denmark (76%, [23]) and Romania (17%, [13]).

Regarding turkey meat contamination, both average level (0.02 ± 0.03 ng/g) and incidence value (30.8%) were higher than the ones determined in pork. However, because turkey meat is eleven times less consumed, the importance of its contamination is accordingly less significant. 

The difference in the reported levels could be explained by the toxicokinetic of OTA, which differs from animal to animal. OTA binding to the serum albumin and recycling in the bile and urine contribute to its general long half-life in animals. However, poultry species appear to eliminate OTA faster than mammals and this leads to a lower OTA accumulation in the blood [93].

No regulatory maximum levels have been established for OTA contamination of meat. However, in view of some individual situations that deserve tighter ruling, some countries themselves have set limits in meat not specified by the EU harmonized guidelines. However, none of the investigated Portuguese meat samples reached levels close to those limits (e.g., Romania 5 ng/g; Italy 1 ng/g) [13,101].

## 4. Exposure Estimate

In a European assessment of the contribution of each food commodity to the mean total dietary intake of OTA [51], cereals and their derived products were considered the major source of human OTA exposure accounting for half of all contributions (50%). In the said study, wine and coffee took a second and third place, contributing about 13% and 10%, respectively. The other contributing food commodities were spices (8%), beer (5%), cocoa (4%), dried fruits (3%) and meat (1%). From the data that Portugal sent to the study, its total population dietary intake was calculated as 0.81 ng/kg.bw/day, being wheat and white (wheat) flour the most contributing of the studied food groups, since they accounted for 0.69 ng/kg bw/day (~85%). The remaining contributions studied were (in ng/kg bw/day) coffee (0.09), wine (0.02) and beer (0.01).

But to assess if this pattern of differential contribution of each food commodity is supported by more recent local and national surveys, a rather simplistic approach can currently be made to compare them. By using different surveys on OTA external dose made at different times, one can estimate the daily intake and therefore confirm or rearrange the order of contribution of each food commodity. One serious drawback is related to the little amount of available data on food contamination, and the fact that the existing data was derived from different analysis procedures, made at different years, which can hinder extensive interpretation efforts. 

To conduct such approach the mean body weight was set at 65 kg, as defined and used by Miraglia and Brera [51], to prevent extra variations of the results. Furthermore, because of the absence of update information related to the mean individual consumption and contamination level some assumptions were needed. 

First of all, to increase reliability, all the consumption data was retrieved from the official institute of statistics (INE; [103]), and all of them correspond to the period/year of sampling and analysis of each commodity. The exception was the value for wheat bread consumption, which was retrieved from the GEMS/FOOD [104] regional *per capita* consumption record, given the absence of a more updated one. It was assumed that maize bread represented a quarter of this consumption, and that in Porto the consumption of the typical maize bread Avintes *broa*, completely substitutes the consumption of normal maize. Secondly, when attributing a contamination level for each commodity, the selection was made considering the one based on the most recent and broad sample volume.

The resulting contributions of each commodity to the total exposure in Portugal through this described evaluation are displayed in Table 1. 

**Table 1:** Individual contributions (%) of each commodity to the EDI (ng/kg.bw/day), according to the corresponding consumption (g(l)/hab/day), average level of contamination (ng/g or ng/mL), and assuming an average body weight of 65 kg, according to recent reports.

					Portuguese population exposure	
Commodity	Consumption	Reference	Average level contamination	Reference	EDI	Contribution (%)
**Cereals**	389.9	[103]	0.64	[54]	3.839	96.47
**Swine meat**	116.44		0.01	[100]	0.018	0.45
**Chicken meat**	60		0		0.000	0
**Turkey meat**	10.7		0.02		0.003	0.08
**Wine**	123.01		0.063	[30]	0.119	3
			**Sum of dietary intake**		3.979	

Comparison of these results with the evaluation data provided for Portugal in the SCOOP report [51] reveals that exposure levels are five times higher in the former (3.98 *vs*. 0.81 ng/kg bw/day), without however surpassing the tolerable estimated intake of 5 ng/kg bw/day, as recommended by the Scientific Committee on Foods—SCF [105]. This difference should however be interpreted taking into due consideration the limitations, one of which is the fact that the food groups included in the estimated average intake for the Portuguese population in the SCOOP report (Table 2; cereal flour, wine, coffee, and beer), are more numerous and representative than the most limited collection available and gathered in the present work (Table 1; cereal grains, meat, and wine). 

Both evaluations share however a similar main contributor, cereals, although their contribution to the total Portuguese estimated intake is higher in the present evaluation (96% *vs.* 85.2%). A possible reason might lie in the fact that in the SCOOP report [51], cereal data only referred to wheat (white) flour. In the present evaluation the cereal data referred to several types of cereal grains, including wheat, maize, oat, rye, barley, *etc*., which are in theory more heavily contaminated than derived cereal products. Cereal flour is the cleanest end product of the milling process, with a low water activity which hinders ochratoxigenic fungal growth. Furthermore, white flour contains much lower concentrations of OTA than whole meal flour because the bran and offal containing high levels of this mycotoxin have been removed.

**Table 2:** Individual contribution (%) of each commodity to the EDI (ng/kg.bw/day), according to the corresponding consumption (g(l)/hab/day), average level of contamination (ng/g or ng/mL), and assuming an average body weight of 65 kg, according to SCOOP report [51].

			Portuguese population exposure	
Commodity	Consumption	Average level contamination	EDI	Contribution (%)
**Cereal (wheat) flour**	235.1	0.19	0.69	84.7
**Coffee**	10.1	0.6	0.09	11.5
**Sweet wine**	1.1	0.01	0.00	0
**Rosé wine**	149.3	0.01	0.02	2.8
**Beer**	177.3	0.003	0.01	1
		**Sum of dietary intake**	0.81	

But the contribution of cereals to the total EDI is even higher when compared to the average estimated intake in the SCOOP report for the European population (50%; [51]), given the importance of cereals in the Portuguese diet, in particular, and the Mediterranean diet, in general. 

Given the importance of bread, in the present evaluation its contribution to OTA intake was also evaluated. The estimated contribution of bread to OTA intake from cereals is 15.10%. Though this value seems unimpressive at first, one must not forget that the cereals make up the remaining 85% include all kinds of cereal, even those not consumed as grain or even as baked goods, such as beer, infant foods, breakfast cereals, *etc*., bringing their individual contributions to the level of, or even below, that of bread.

This value supports the major contribution of bread, in particular, and cereals, in general, to OTA exposure. Previously both the SCOOP report [51] and a French total diet study [106] demonstrated that cereals and cereal derivatives are the food groups contributing the most to OTA exposure. In the former, cereals and their derivatives accounted for 50% and in the latter, cereals and cereal products (bread, rusk, breakfast cereals, pasta, rice and semolina, other cereals, Viennese bread, biscuits and cakes) for 70%, and bread alone corresponded to almost 33%. 

It is also evident from Table 3 that although maize bread features higher levels of contamination, the much higher consumption of wheat bread results in the exposure of Portuguese citizens to higher OTA levels through the consumption of the latter. For all the population exposure wheat bread accounted for 74.2% alone, whereas maize bread contributed with 25.8%. In the specific case of Porto, where normal maize bread consumption is replaced with by Avintes maize bread, wheat bread contribution lowers (65.9%) and comes closer to the contribution of Avintes *broa* (34.1%). This is due to a fairly high contamination of Avintes bread when compared to normal maize bread as described above. In fact, this higher contamination of Avintes maize bread, increases the sum of the dietary intake among Porto’s population, against the total population. Therefore, a Porto resident is more exposed to OTA through bread consumption, specifically through ingestion of Avintes maize bread.

**Table 3:** Contribution (%) of the different types of bread (wheat, normal maize, Avintes maize bread) to the EDI (ng/kg.bw/day), according to the corresponding consumption (g/hab/day; [104]), average level of contamination (ng/g), and assuming an average body weight of 65 kg.

				Portuguese population exposure		Porto population exposure	
Bread type	Consumption	Average level contamination	References	EDI	Contribution (%)	EDI	Contribution (%)
Wheat bread	117.2	0.238	[45,67,71]	0.430	74.17	0.430	65.88
Maize bread	29.3	0.332	[45,67]	0.150	25.83	-	-
Avintes bread*	29.3	0.494	[67]	-	-	0.223	34.12
				0.580	**Sum of dietary intake**	0.653	**Sum of dietary intake**

* Applicable to Porto only, in replacement of the consumption of normal maize bread.

Furthermore, the EDIs from bread (ng/kg bw/day) reported for both the entire population (0.580) and the one from Porto (0.653) are lower, but close to those recorded by Leblanc *et al*. [106] for the French population (0.71), and by the SCOOP report for Spanish inhabitants (0.77) [51]. Conversely, the values of the EDI in the present study are higher than those calculated for German (0.36) inhabitants and higher than the calculated ones for Danish residents (0.19). 

As for the second place, although distant from bread, the most contaminated food commodity, wine, contributes to 3% of OTA exposure. Although the levels detected in the surveys among Portuguese wine are low, the high *per capita* consumption (4th place in the World) adds importance to this commodity among Portuguese citizens. In fact, this contribution is lower than the one of the average European population (13%) but close to the estimated for the Portuguese population (2.5%) by the SCOOP report [51]. In the French study, wine and other grape products, along with coffee, nuts and oilseeds contributed less than 5% to the total exposure [106]. Furthermore, the low levels encountered in Portuguese wine surveys also mean that for other populations the degree of exposure will be necessarily inferior. So, in conclusion, the level of contribution to other countries’ consumers by this most appreciated product of export does not stand at the same level. 

The third place goes to the swine meat. Again, and even more pronounced than wine, the high *per capita* pork consumption compensated the low levels of contamination encountered (0.01 ng/g). However and apparently its contribution to the OTA exposure does not merit the same concern as cereals. Turkey meat, although with a higher contamination level, because so much less consumed it presents an even lower risk to the Portuguese consumers (0.08% of total OTA intake). 

The herein estimated sum of dietary intake (3.979 ng/kg bw/day) is inferior, although close to the tolerable daily intake (TDI) as recommended by the European Commission’s SCF, 5 ng/kg bw/day [105], and far below the ones proposed by the Joint FAO/WHO Experts Committee on Food Additives, 14.28 ng/kg bw/day [107] and EFSA, 17.14 ng/kg bw/day [108] but is over the virtual safety dose of 1.5 ng/kg bw/day calculated by Kuiper-Goodman and Scoot [109] and very close to the TDI of 4 ng/kg bw/day as recently calculated by Kuiper-Goodman *et al*. [89].

Furthermore, the fact that the majority of the calculated intakes are inferior to the tolerable doses should not be neglected. First because between regions/cities there may exist wide variations in consumption of the more risky foods, and so if calculation is based on an average value that means that some individuals may exceed this value, and so be at risk. Second, individuals may differ in their sensitivity to OTA. Finally, this mycotoxin may be additive to, or synergistic with, other chemicals in food and the environment [15,21,24,110,111].

It is also important to underline that the exposure through external dose, as used above, tends to be less accurate due to the fact that only an estimation can be made regarding the eating habits of a population, an estimation that, moreover, results in an average value, failing to account for those who would fall in the high end of the contamination scale—exactly those who are in greater risk [42]. So the information given through internal dose assessment is also important to the estimation of daily intake, on the basis of OTA level in serum/plasma. The main advantages associated are: (1) it is not necessary to be aware of the source of contamination involved—ingestion of contaminated food or inhalation of contaminated air; and (2) it requires a single determination *per* person, and saves all the problems associated with the food sampling methods and consumption data collection. It can however confer an underestimation of the intake, given the approximations in respect to toxicokinetic properties and the assumptions from which it stands [111,113]. 

The equation that allows the estimation based on plasma concentration is the Klaassen equation, and so was the one used in the only study performed on blood from healthy central Portuguese inhabitants [33]. In this study the estimated daily intake (EDI; ng/kg bw/day) was 0.56 in Coimbra (range 0.26–1.29), 0.59 in Ereira (range 0.19–2.56), and 1.05 in Verride (range 0.34–3.35), all of them below the tolerable daily intake proposed values. If the results from other studies are brought into the comparison, one can see that the Portuguese value falls into the range between the EDI reported in Japan (0.08; [50]) and the one reported in Lleida, Spain (1.69; [113]). The Portuguese reported EDI, did not surpass any of the several recommended TDIs, and so is not considered to represent a health hazard.

## 5. Conclusions

Through this comprehensive review on the reports on OTA occurrence in food and biological fluids, several conclusions can be drawn. First, a widespread occurrence of OTA is evident, as confirmed by the high prevalence in biological fluids and some foodstuffs, especially in the cereal and cereal based foods, like bread, which coupled with their high consumption contributes to a higher OTA exposure. The reported levels for other commodities are several times lower, and simply reach significance because of a high consumption, as in the case of pork and wine. 

The rare occurrence of limit-surpassing samples indicates that in general the exposure to this mycotoxin is unlikely to pose a threat to consumer health, although a continuous intake at low levels can still carry risks. Therefore, the widespread foodstuffs occurrence and the favorable environmental conditions in Portugal encourage a regular testing to monitor the situation and protect consumer health. 
